# Determination of Heavy Metals Concentration in Traditional Herbs Commonly Consumed in the United Arab Emirates

**DOI:** 10.1155/2015/973878

**Published:** 2015-04-27

**Authors:** Rania Dghaim, Safa Al Khatib, Husna Rasool, Munawwar Ali Khan

**Affiliations:** Department of Natural Science and Public Health, College of Sustainability Sciences and Humanities, Zayed University, P.O. Box 19282, Dubai, UAE

## Abstract

Herbs are extensively consumed in the United Arab Emirates for their flavoring and medicinal properties. This study aimed at determining the concentration of heavy metals in selected traditional herbs consumed in the United Arab Emirates (UAE). A total of 81 samples of seven herbs, parsley (*Petroselinum crispum*), basil (*Ocimum basilicum*), sage (*Salvia officinalis*), oregano (*Origanum vulgare*), mint (*Mentha spicata*), thyme (*Thymus vulgaris*), and chamomile (*Matricaria chamomilla*), were purchased from the local market in Dubai and analyzed for their cadmium, lead, copper, iron, and zinc contents. Microwave-assisted digestion was applied for the dissolution of the samples and heavy metals concentration was determined using Atomic Absorption Spectrometry (AAS). Metals were found to be present in varied concentrations in the herb samples. The concentration ranges were found as follows: less than 0.1–1.11 mg·kg^−1^ for cadmium, less than 1.0–23.52 mg·kg^−1^ for lead, 1.44–156.24 mg·kg^−1^ for copper, 12.65–146.67 mg·kg^−1^ for zinc, and 81.25–1101.22 mg·kg^−1^ for iron. The findings of the study suggest that most of the analyzed herbs contained unsafe levels of heavy metals that exceeded the World Health Organization (WHO) permissible limits (PL).

## 1. Introduction

Herbal remedies are widely used for the treatment of various illnesses. They often contain highly active pharmacological components including minerals and trace metals [1]. Herbal plants represent an important class of various traditional medicine systems and, in recent years, they are increasingly used in the primary health care intervention in both developed and developing countries. According to World Health Organization (WHO) estimates, nearly 70–80% of the world population still primarily relies on nonconventional medications, mostly derived from herbal plants [2, 3]. The United Arab Emirates (UAE) is known for its rich tradition of herbal medicine, and the Emirati population is quite familiar with the medicinal and flavoring properties of several herbs. Herbs are traditionally used for the treatment and prevention of ailments such as stomach pain, headache, diabetes, hypertension, rheumatism, and many others [4]. The hyperarid environment of the UAE hosts a diverse range of medicinal plants. According to a recent study on the diversity and conservation status of plants in the UAE, 18% of the plant species were found to possess medicinal properties [4]. A survey on the herbal remedies used in Abu Dhabi has found 65 different herbs used to treat 48 conditions [5].

In parallel with the increasing interest in the therapeutic benefits of herbal products, there has been an increasing concern over the safety and toxicity of natural herbs and formulations available on the market. There is a widespread misconception that natural herbs and plants are inherently safe; nevertheless, there has been a large volume of reports on incidences of toxicity and adverse effects linked to the use of herbal plants and their formulations in different parts of the world [6]. In a survey on herbal remedies used in the UAE, the majority of respondents felt they were safe; however, 27 people reported adverse health effects [5]. The toxicity of herbal plants may be related to contaminants such as pesticides, microbes, heavy metals, chemical toxins, and adulterants [7]. In general, the geography, the geochemical soil characteristics, contaminants in the soil, water, and air, and other growth, transport, and storage conditions can significantly affect the properties and the quality of the herbal plants and their formulations [7].

The toxicity of trace metals on human health and the environment has attracted considerable attention in recent years. Plants are the main link in the transfer of heavy metals from the contaminated soil to humans. Heavy metals have a tendency to accumulate in the food chain. Heavy metals have low excretion rates through the kidney which could result in damaging effects on humans even at very low concentrations. Metals such as zinc, copper, iron, manganese, and chromium are essential nutrients; they are important for the physiological and biological functions of the human body. However, an increase in their intake above certain permissible limits can become toxic [8, 9]. In general, a number of health problems were linked to excessive uptake of dietary heavy metals including a decrease in immunological defenses, cardiac dysfunction, fetal malformation, impaired psychosocial and neurological behavior, gastrointestinal cancer, and many others [10, 11]. The heavy metal contamination of herbal remedies has been reported earlier in several Asian, South American, and African herbal products in different countries [12–16].

There is very little information available on the safety of traditional herbs and their products sold in the UAE market. This study aims at determining the level of heavy metal contamination in some commonly consumed herbs to assess their relative safety and potential health risks based on the World Health Organization (WHO) standard limits.

## 2. Materials and Methods

A total of eighty-one herb samples both local and imported (fresh and dried) of seven different types of herbs were purchased from 13 different sources in the local markets of Dubai. The herb samples used in this study were purchased from authenticated shops/supermarkets approved by Dubai municipality.  Table 1 shows a list of the herbs common and scientific names and the number of samples analyzed. Fresh samples were air-dried at room temperature. All samples were powdered, sieved, and stored in plastic bags for metal analysis.

All glassware and digestion vessels were soaked in 20% nitric acid and rinsed with ultrapure water (Millipore Elix Advantage Water Purification System, Millipore, MA, USA). Multielement standard solutions of lead (Pb), cadmium (Cd), iron (Fe), zinc (Zn), and copper (Cu) were prepared by dilution of 1000 mg/L stock solutions (Fluka TraceCert Ultra, Sigma-Aldrich) with 5% nitric acid (HNO_3_) solution. The calibration curve for each element was linear and a correlation coefficient of 0.995 was obtained.

For microwave digestion, 0.5 g of the herb sample was accurately weighed into a digestion vessel (MARSXpress), followed by addition of 0.5 mL of 37% hydrochloric acid (HCl) (trace metal concentrated, Suprapur, Merk), 9.0 mL of 69% nitric acid (HNO_3_) (trace metal concentrated, Suprapur, Merk), and 1 mL of 30% hydrogen peroxide (H_2_O_2_) (Sigma-Aldrich). The mixture was subjected to US-EPA 3052 microwave-assisted digestion in MARS Microwave digestion system (CEM Corporation, Matthews, USA) [17]. At the end of the digestion program, the samples were filtered and quantitatively transferred to 25 mL volumetric flask and diluted with 0.2% nitric acid solution. The concentration of metals in the sample was determined using atomic absorption spectrophotometer (AA240FS series by Varian Australia Pty Ltd.).

All quality control and assurance measures were taken including calibration check measures, determination of Method Quantification Limits (MQL), and replicate analysis of samples. Concentration of heavy metals is expressed as the mean value (mg·kg^−1^) of dry weight ± SD of three subsamples collected from the same source.

## 3. Results and Discussion

The results of heavy metals analysis in the selected samples are presented in Table 2.

### 3.1. Cadmium

The cadmium (Cd) concentration varied from less than 0.1 to 1.11 mg·kg^−1^. Cadmium was not detected in any of the thirteen mint samples. The maximum concentrations of Cd detected in parsley, chamomile, basil, sage, oregano, and thyme were 0.21, 0.82, 1.11, 0.88, 0.35, and 0.63 mg·kg^−1^, respectively.

Overall, among the 78 herb samples analyzed for cadmium, 29% of the samples contained high levels of cadmium exceeding 0.3 mg·kg^−1^, the permissible limit (PL) set by FAO/WHO for medicinal herbs and plants in different countries [18, 19]. The cadmium levels in all mint and parsley samples were below the permissible limit (PL). 55%, 80%, 66%, 9%, and 27% of chamomile, basil, sage, oregano, and thyme samples, respectively, exceeded the permissible limits (PL) for cadmium in medicinal herbs and plants. Similar results of high levels of cadmium in Egyptian and Iranian medicinal herbs and plants have been reported in earlier studies [20, 21].

The high levels of cadmium possess a serious toxicological effect on human health. Kidney is the critical target organ in the exposed population. Excretion of cadmium is very slow and it accumulates in human kidney for a relatively long time, resulting in an irreversible impairment of the renal tract [22–24]. At high concentrations, cadmium produces serious effects on the liver and vascular and immune system [24].

### 3.2. Lead

The content of lead (Pb) in the analyzed samples ranged from less than 1.0 to 23.52 mg·kg^−1^. The maximum concentrations of lead in mint, parsley, chamomile, basil, sage, oregano, and thyme were found to be 9.24, 12.83, 11.40, 16.15, 21.76, 18.06, and 23.52 mg·kg^−1^, respectively.

The FAO/WHO maximum permissible limit of lead in consumed medicinal herbs is 10 mg·kg^−1^ [18, 19]. The obtained results showed that 64% of the analyzed samples exceeded this limit. The levels of lead in all mint samples were below the permissible limits (PL) and ranged between 1.44 and 9.24 mg·kg^−1^. However, 46%, 44%, 90%, 100%, 90%, and 91% of parsley, chamomile, basil, sage, oregano, and thyme, respectively, showed concentrations of lead higher than 10 mg·kg^−1^.

High concentration of lead above permissible limits in medicinal plants and herbs has been reported in other Middle Eastern countries. The mean lead concentration in most commonly and commonly used medicinal herbs in Jordan was found to be 13.9 and 13.1 mg/kg on a dry weight basis, respectively [23]. Maximum levels of 14.4 mg/kg and 21.7 of lead were also reported in Egyptian and Iranian spices and medicinal plants, respectively [20, 21].

Lead is known to be one of the highly toxic environmental pollutants. It can complex with various biomolecules and adversely affect their functions. Lead exposure may have an adverse effect on the blood, nervous, immune, renal, skeletal, muscular, reproductive, and cardiovascular systems causing poor muscle coordination, gastrointestinal symptoms, brain and kidneys damage, hearing and vision impairments, and reproductive defects [25, 26]. Exposures to lead at early childhood and prenatally are associated with slowed cognitive development, learning deficits, and many other effects [25, 26].

### 3.3. Copper

The copper (Cu) concentrations varied in a wide range between 1.44 and 156.24 mg·kg^−1^. The maximum concentrations of copper in mint, parsley, chamomile, basil, sage, oregano, and thyme were 12.32, 13.29, 12.99, 18.87, 156.24, 41.64, and 13.16 mg·kg^−1^, respectively.

The regulatory limits of the WHO/FAO have not been established yet for the copper in herbal medicines [27]. China and Singapore had set limits for copper in medicinal plants at 20 and 150 mg·kg^−1^, respectively [27]. In this study, only 12% of the samples exceeded the Chinese limit of 20 mg·kg^−1^, where one sage sample showed a value above 150 mg·kg^−1^ limit of Singapore. 66% of the chamomile samples and 18% of each of the sage and oregano samples exceeded 20 mg·kg^−1^.

Copper is an essential component of many enzymes, therefore playing a significant role in a wide range of physiological processes including iron utilization, free radicals elimination, bone and connective tissues development, melanin production, and many others. Nevertheless, excessive intake of copper can cause dermatitis, irritation of the upper respiratory tract, abdominal pain, nausea, diarrhea, vomiting, and liver damage [23, 27].

### 3.4. Zinc

The concentration of zinc (Zn) in the analyzed samples ranged between 12.65 and 146.67 mg·kg^−1^. The maximum concentrations of zinc in mint, parsley, chamomile, basil, sage, oregano, and thyme were 52.97, 50.10, 38.93, 112.19, 58.78, 37.28, and 146.67, respectively. Overall, results revealed that only 19% of the samples analyzed had concentrations higher than 50 mg·kg^−1^, the FAO/WHO permissible limit (PL) set for zinc in herbal medicines [18, 19]. Out of the seven different herbs analyzed, 7% of each of mint and parsley samples, 36% of sage, and 50% of basil samples exceeded the permissible limits for zinc.

Zinc is an essential trace element necessary for proper growth, blood clotting, thyroid function, and protein and DNA synthesis. Little information is available on Zn toxicity; however, high zinc intake beyond permissible limits produces toxic effects on the immune system, blood lipoprotein levels, and copper level [28].

### 3.5. Iron

The observed range of iron (Fe) in the current study was found between 81.25 and 1101.22 mg·kg^−1^. The maximum concentrations of iron in mint, parsley, chamomile, basil, sage, oregano, and thyme were 821.01, 605.50, 581.30, 1101.23, 799.31, 420.52, and 764.51 mg·kg^−1^, respectively.

The WHO limit for iron in medicinal herbs has not been established yet. The results of the current study show a wide variation of iron in different herb samples. These results are comparable to values of iron found in Egyptian spices and medicinal plants that ranged between 26.96 and 1046.25 mg·kg^−1^ [20].

Iron has several key functions in the human body including oxygen supply, energy production, and immunity. Iron overdose is associated with symptoms of dizziness, nausea and vomiting, diarrhea, joints pain, shock, and liver damage. Iron toxicity has an adverse effect on various metabolic functions and cardiovascular system [23].

Overall the results of analysis showed that heavy metals were present in varied concentrations in the seven traditional herbs commonly consumed in the UAE. The concentration of heavy metals in many cases exceeded the internationally accepted permissible levels. The wide variations in metal concentrations in the analyzed herbs could be attributed to differences in the plant metal uptake and translocation capabilities. Metal uptake by plants depends on several factors including the plant species and their stage of growth, the soil type, and the type of metals absorbed [29, 30]. Studies have shown wide variations in concentration factor of different metals among different plant species and sampling sites. For example, high transfer values of cadmium, copper, and nickel from soils irrigated with wastewater to food crops were observed, indicating a stronger accumulation of these metals by the food crops compared to other metals [31]. Studies have also shown that plants do not accumulate lead. The concentration of lead in plants was found to be more correlated to the level of lead in the atmosphere [29, 31]. Another study that monitored the metallic micronutrients and heavy metals in herbs, spices, and medicinal plants from Austria has found that species such as St. John's wort, poppy, yarrow, chamomile, and absinth have higher tendency to accumulate cadmium. Similarly, results of this study have shown higher cadmium levels in chamomile, in addition to basil and sage [32]. The bioavailability of metals is influenced by several factors among which are the soil pH, the metal levels in the soil, the oxidation reduction potential of the soil, and other chemical and physical factors [29, 30]. Furthermore, contamination could occur during storage and/or at the point of sale. A study was conducted in India to compare the heavy metals concentration in different* Berberis* species collected from their natural habitats and their market samples. Results have shown that market samples were more contaminated with heavy metals than natural samples [33]. In general, herbs can be contaminated during growing, harvesting, and processing. Sources of heavy metal contamination in herbs could be linked to water used in irrigation, polluted soils, fertilizers and pesticides, industrial emissions, transportation, and harvesting and storage processes. The health risk due to metal contamination, in general, depends on the average daily dietary intake.

## 4. Conclusion

In conclusion, the results of this study indicate a potential health risk of heavy metals to consumers in the UAE over long-term consumption of contaminated herbs. The findings of this study also highlight the significance of safety and hygiene practices and measures starting from the harvest area of the herbs until they reach the consumer end. It is evident that there is an urgent need to implement a regular monitoring and testing program on the quality of the local and imported herbs sold in the UAE market. Further studies are required to determine the presence of toxic metals and to assess their long-term cumulative risk on consumer health.

## Figures and Tables

**Table 1:** Herbs common name and scientific name and the number of collected samples.

Common name	Scientific name	Total number of samples collected (*N* = 81)
Parsley	*Petroselinum crispum *	13
Basil	*Ocimum basilicum *	11
Sage	*Salvia officinalis *	11
Oregano	*Origanum vulgare *	11
Mint	*Mentha spicata *	13
Thyme	*Thymus vulgaris *	13
Chamomile	*Matricaria chamomilla *	9

**Table 2:** Concentration range of cadmium, lead, copper, iron, and zinc (mg·kg^−1^) in herb samples, and percentage of samples above permissible limits (PL).

Herbs		Cadmium (Cd)	Lead (Pb)	Copper (Cu)^∗^	Iron (Fe)	Zinc (Zn)
Mint	Range (mg·kg^−1^)	∗∗	1.44 ± 0.67–9.24 ± 1.02	3.82 ± 0.02–12.32 ± 0.31	150.57 ± 10.34–821.02 ± 17.83	12.65 ± 1.05–52.97 ± 2.81
	% of sample above PL (*n*/*N*)	0% (0/13)	0% (0/13)	0% (0/13)	ND	7% (1/13)

Parsley	Range	^∗∗^−0.21 ± 0.07	^∗∗^−12.83 ± 0.97	4.21 ± 0.13–13.29 ± (0.29)	81.26 ± 7.00–605.50 ± 14.87	19.32 ± 0.35–50.10 ± 1.22
	% of sample above PL (*n*/*N*)	0% (0/13)	46% (6/13)	0% (0/13)	ND	7% (1/13)

Chamomile	Range	^∗∗^−0.82 ± 0.02	5.37 ± 1.25–11.40 ± 1.32	6.12 ± 1.03–12.99 ± 0.48	188.27 ± 3.72–581.30 ± 41.889	25.23 ± 3.56–38.93 ± 3.14
	% of sample above PL (*n*/*N*)	55% (5/9)	44% (4/9)	66% (6/9)	ND	0% (0/9)

Basil	Range	0.13 ± 0.01–1.11 ± 0.07	9.01 ± 0.28–16.15 ± 2.53	1.44 ± 0.53–18.87 ± 1.46	185.73 ± 13.02–1101.23 ± 49.46	15.22 ± 1.31–112.19 ± 3.44
	% of sample above PL (*n*/*N*)	80% (8/10)	90% (9/10)	0% (0/10)	ND	50% (5/10)

Sage	Range	^∗∗^−0.88 ± 0.08	12.66 ± 1.54–21.76 ± 1.33	5.17 ± 0.03–156.24 ± 1.22	204.15 ± 19.62–799.31 ± 36.85	24.64 ± 0.53–58.78 ± 2.72
	% of sample above PL (*n*/*N*)	66% (6/9)	100% (10/10)	18% (2/11)	ND	36% (4/11)

Oregano	Range	^∗∗^−0.35 ± 0.02	9.39 ± 1.77–18.06 ± 2.32	3.99 ± 0.35–41.64 ± 2.20	155.74 ± 4.67–420.52 ± 15.19	13.33 ± 2.15–37.28 ± 0.71
	% of sample above PL (*n*/*N*)	9% (1/11)	90% (10/11)	18% (2/11)	ND	0% (0/11)

Thyme	Range	^∗∗^−0.63 ± 0.07	9.07 ± 0.34–23.52 ± 1.68	3.52 ± 0.22–13.16 ± 0.33	120.75 ± 1.82–764.51 ± 39.15	16.50 ± 1.29–146.67 ± 7.57
	% of sample above PL (*n*/*N*)	27% (3/11)	91% (11/12)	0% (0/12)	ND	38% (5/13)

% of all samples above PL		29%	64%	12%	ND	19%

^∗^China set PL 20 mg·kg^−1^.

^**∗∗**^Concentration below Method Quantification Limit (MQL): 0.1, 1 mg·kg^−1^ for cadmium and lead, respectively.

*N*: total number of samples analyzed for the metal; *n*: number of samples above permissible limits (PL).

ND: set limits not determined for iron.
